# Supplementary material for: Disruption of trait-environment relationships in African megafauna occurred in the middle Pleistocene
Source: Nat Commun. 2023 Jul 18;14:4016. doi: 10.1038/s41467-023-39480-8 (PMC10354096; doi:10.1038/s41467-023-39480-8)
Supplement: Supplementary file 1 — Supplementary Information [file 41467_2023_39480_MOESM1_ESM.pdf]

Supplementary Information for

Disruption of trait-environment relationships in African megafauna occurred in the middle Pleistocene

Daniel A. Lauer*, A. Michelle Lawing, Rachel A. Short, Fredrick K. Manthi, Johannes Müller, Jason J. Head, Jenny L. McGuire

*Daniel A. Lauer
E-mail: lauerd@gatech.edu

Supplementary Figures

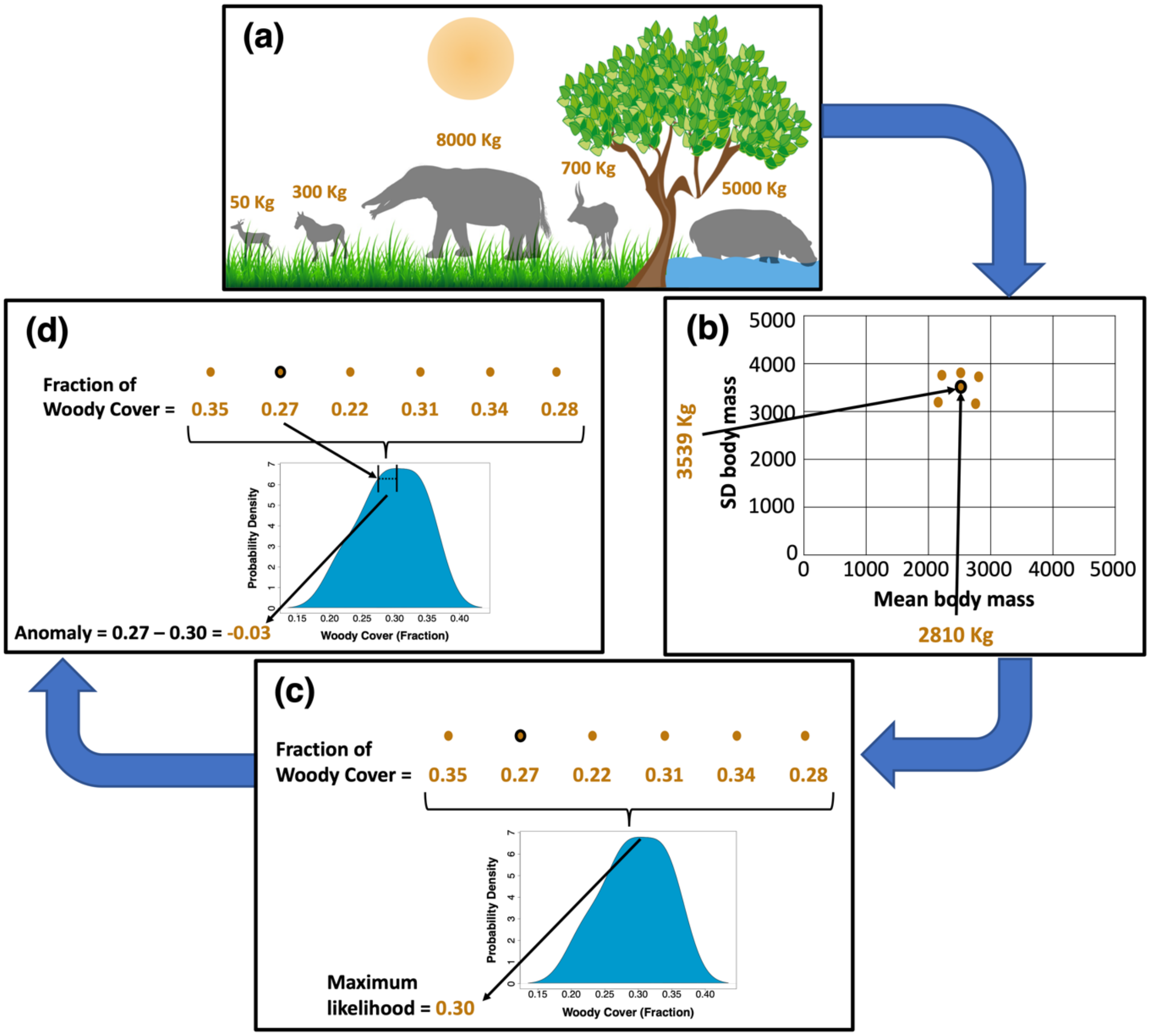

Supplementary Figure 1. Conceptual representation of the ecometric analysis framework. (a) Consider a community/site of large herbivores composed of five total species. (b) The site is binned into a grid based on the mean and standard deviation of the body masses of its five species. In the same trait bin as our target site, we also see five other sites representing communities of large herbivores from across eastern Africa and across all points in time. (c) A probability density function (PDF) of the fractions of woody cover at the six total sites is plotted, from which the maximum likelihood woody cover for that set of sites is determined (Supplementary Figure 3 shows the maximum likelihood values for complete grids). (d) Then, the ecometric anomaly of our target site is calculated as the difference between its measured and its maximum-likelihood-estimated woody cover. This calculation is repeated across all sites in all trait bins, and the process is repeated for all traits such that each site is associated with an anomaly value for each trait. Gold coloring denotes data points and important numerical values.

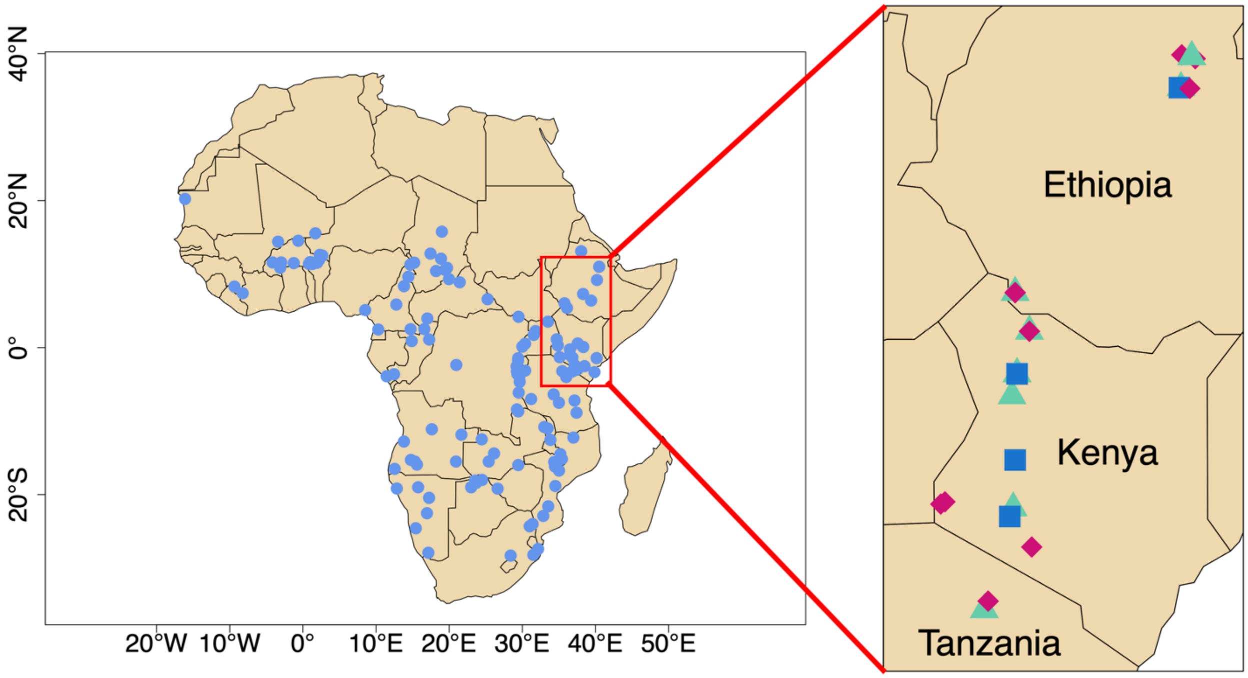

Supplementary Figure 2. Maps of the locations of the fossil and modern communities/sites of large herbivores analyzed in this study. The fossil sites span Ethiopia, Kenya, and Tanzania, and are color-coded by those representing the Miocene (blue square), Pliocene (aqua triangle), Pleistocene (pink diamond), and Holocene (purple circle) epochs. The modern sites span national parks, games reserves, and protected areas throughout all of Africa. Axes in the plot refer to latitude and longitude coordinates. Maps were made with Natural Earth (free vector and raster map data @ naturalearthdata.com).

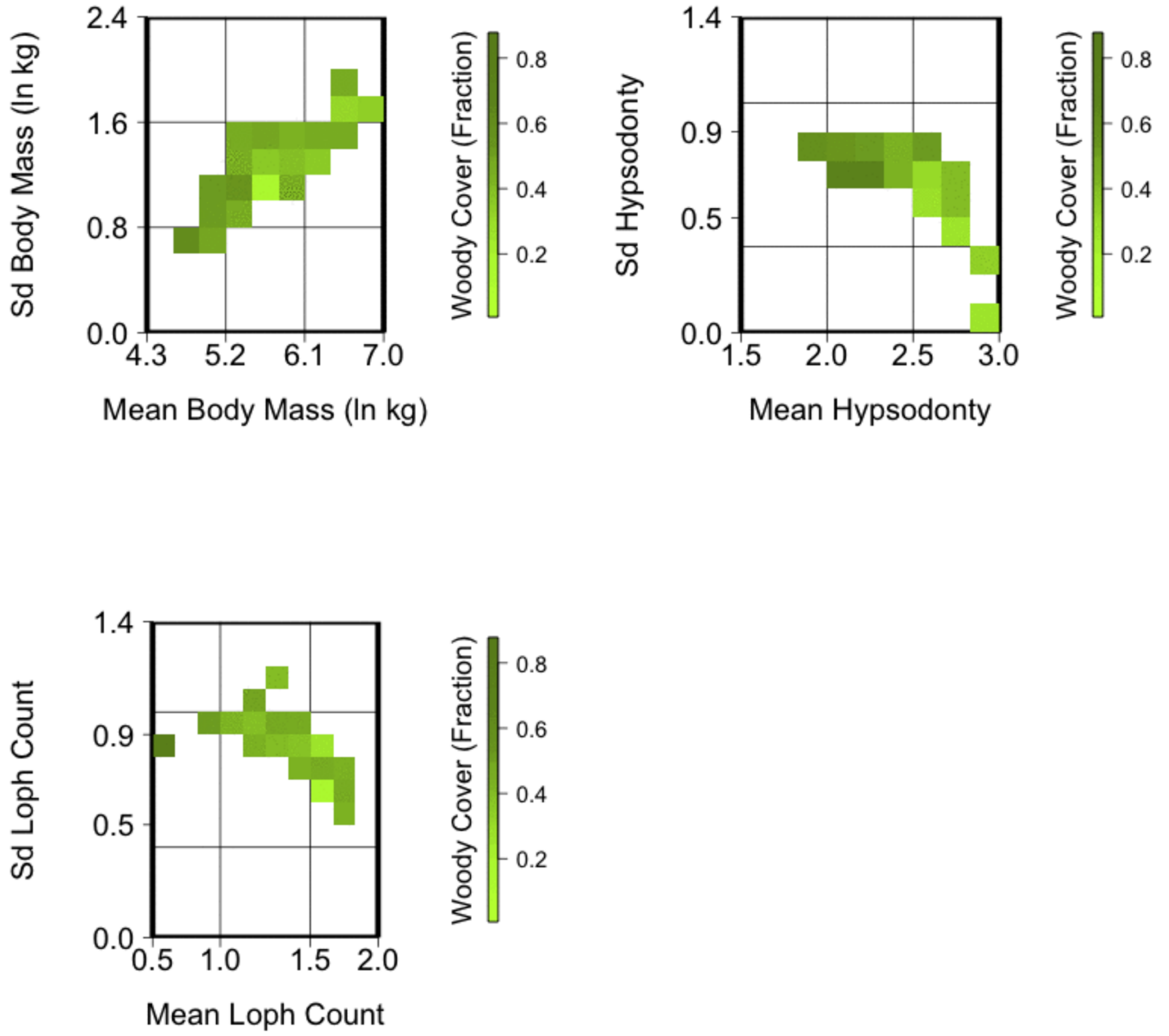

Supplementary Figure 3. Maximum likelihood fraction of woody cover values for groups of trait bins, stemming from ecometric models built with respect to body mass, hypsodonty, and loph count traits. Each bin contains a group of communities of large herbivores. Communities were sorted into the bins based on the mean and standard deviation of the trait values of their component species. The maximum likelihood woody cover of each bin is the peak of the probability density function of the woody cover values of all sites comprising the bin. The number of bins per axis was determined using the Scott optimization method, based on the distribution of values along the metric representing the axis (see Methods). Body mass is measured in log-transformed kg, hypsodonty on a scale of 1 to 3 (1 = brachydont, 2 = mesodont, 3 = hypsodont), and loph count as a discrete measure from 0 to 2. Source data are provided as a Source Data file.

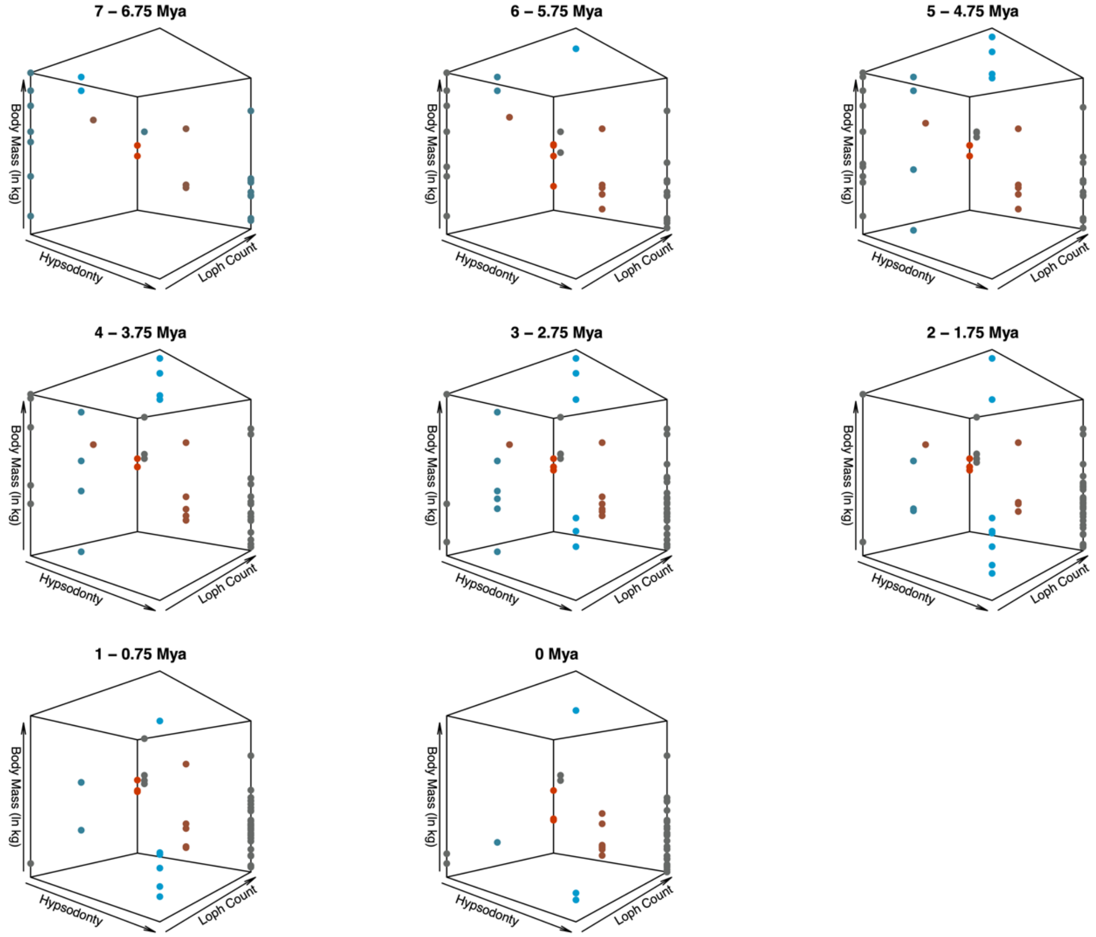

Supplementary Figure 4. Examples of the three-dimensional plots used to calculate functional diversity for each time bin in Figure 1d. These plots were sampled every 1 Ma, each representing a 250,000-year time bin. In each plot, the axes refer to body mass, hypsodonty, and loph count. Each data point refers to a species, positioned based on its values for these three traits, and color-coded by its depth in the plot: data points closer to red in color are located deeper within the plot's three-dimensional space, while those closer to blue are located closer to the front face of the space. For each time bin, we calculated functional diversity as the mean of the Euclidean distances of the data points to their collective centroid in the three-dimensional space. Source data are provided as a Source Data file.

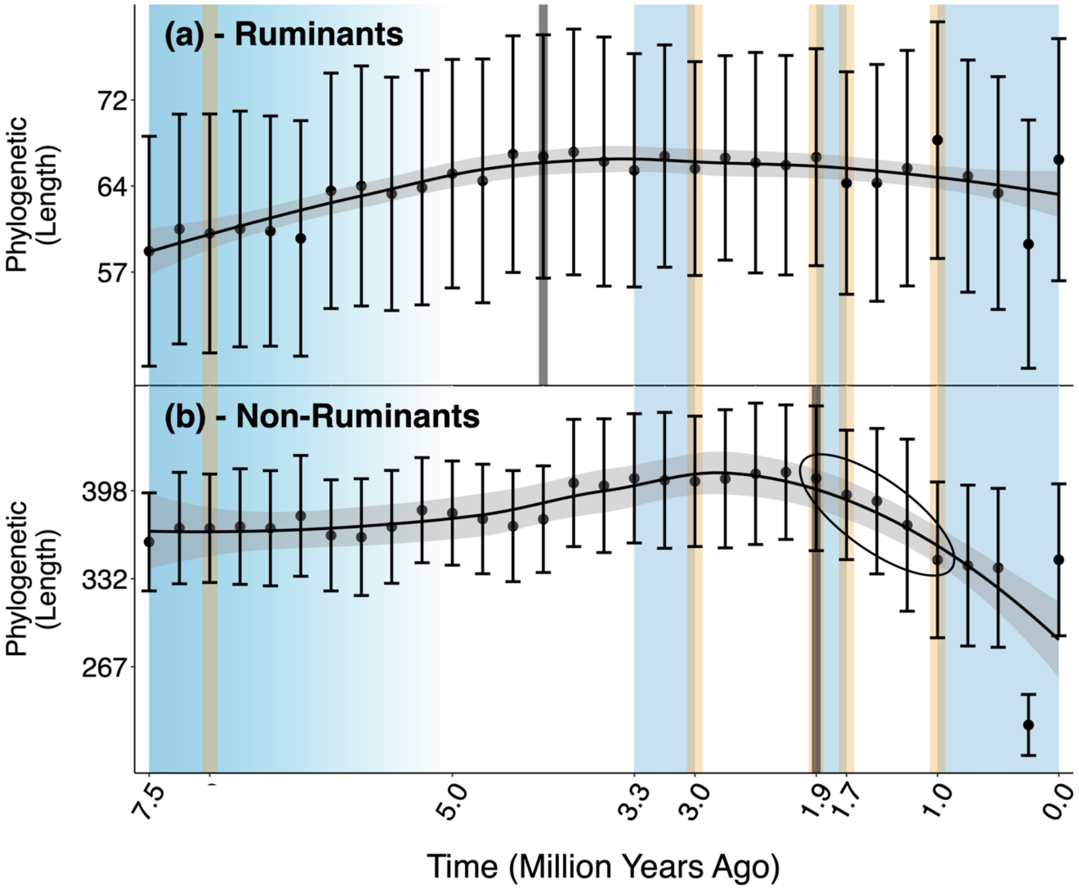

Supplementary Figure 5. LOESS regression trends in phylogenetic diversity of herbivorous megafauna in eastern Africa, performed separately for ruminants (a) and non-ruminants (b). Each data point represents a 250,000-year time bin. Among the species of megafauna occurring in each time bin, phylogenetic diversity is measured as the sum of the branch lengths (in millions of years) of the phylogenetic tree connecting their genera. Each data point is presented as a mean value $\pm$ one standard error from n=1,000 independent samples of genera (see Methods). LOESS regression curves use a smoothing parameter of 0.75. Faded gray bars denote breakpoints from breakpoint analysis. Blue shaded areas refer to events related to environmental change and orange to events in hominin evolution. These events are encompassed in key time intervals, as follows: 7.5-5 Ma includes the onset of grassland expansion and the emergence of hominins (7 Ma); 3.3-3 Ma includes the mid-Pliocene Warm Period and the development of Oldowan hominin tools (3 Ma); 1.9-1.7 Ma includes the increase in climate variability and aridity, as well as the emergence of *Homo erectus* (1.9 Ma) and their development of Acheulean technology (1.7 Ma); and $\leq$1 Ma includes the intensification of periods of aridity, as well as rapid cranial growth in hominins (1 Ma). Circled points in (b) reference the most dramatic decline in phylogenetic diversity, equivalent in timing to those circled in Figure 1c. This analysis was performed separately for ruminants and non-ruminants to address their different biodiversity patterns over the period in which phylogenetic diversity was in decline [1]. Source data are provided as a Source Data file.

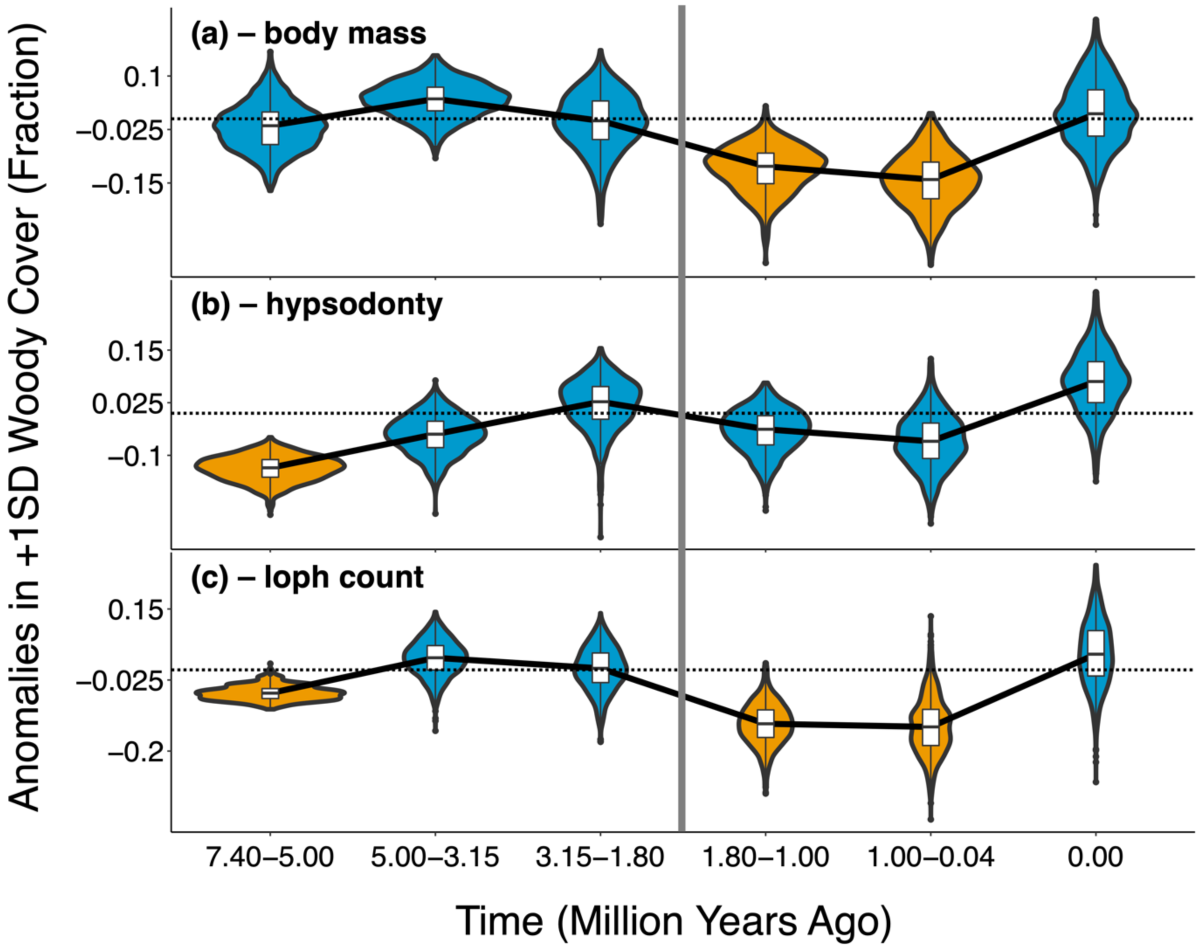

Supplementary Figure 6. Trends in the ecometric anomalies (see Supplementary Figure 1d) of eastern African communities of large herbivores, with respect to estimations of communities' mean + 1SD woody cover values from body mass (a), hypsodonty (b), and loph count (c). This figure differs from Figure 2 in that it refers to estimations of communities' woody cover values that are one standard deviation above their means (see Methods). Maximum likelihood estimations of woody cover were made for each community using an ecometric model of all communities together through time. Each violin plot depicts the distribution of n=1,000 independent samples, where each sample is the mean ecometric anomaly of a random subset of communities occurring within the plot's time bin (x-axis). Each box plot depicts its distribution's median (center) and interquartile range (bounds of box), plus 1.5 * the interquartile range above and below the box (whiskers). Dotted lines indicate a mean anomaly of zero. Blue plots represent distributions whose 95% confidence intervals contain zero, while orange plots represent those whose confidence intervals do not. The vertical gray line represents the point at which ecometric anomalies shifted significantly after a long period of consistency. Time bins on the x-axis are based on the cutoff points of the time intervals depicted in Figure 1. 3.15 Ma is the midpoint of 3.3-3 Ma, 1.8 Ma is the midpoint of 1.9-1.7 Ma, and the final time bin represents the present. Source data are provided as a Source Data file.

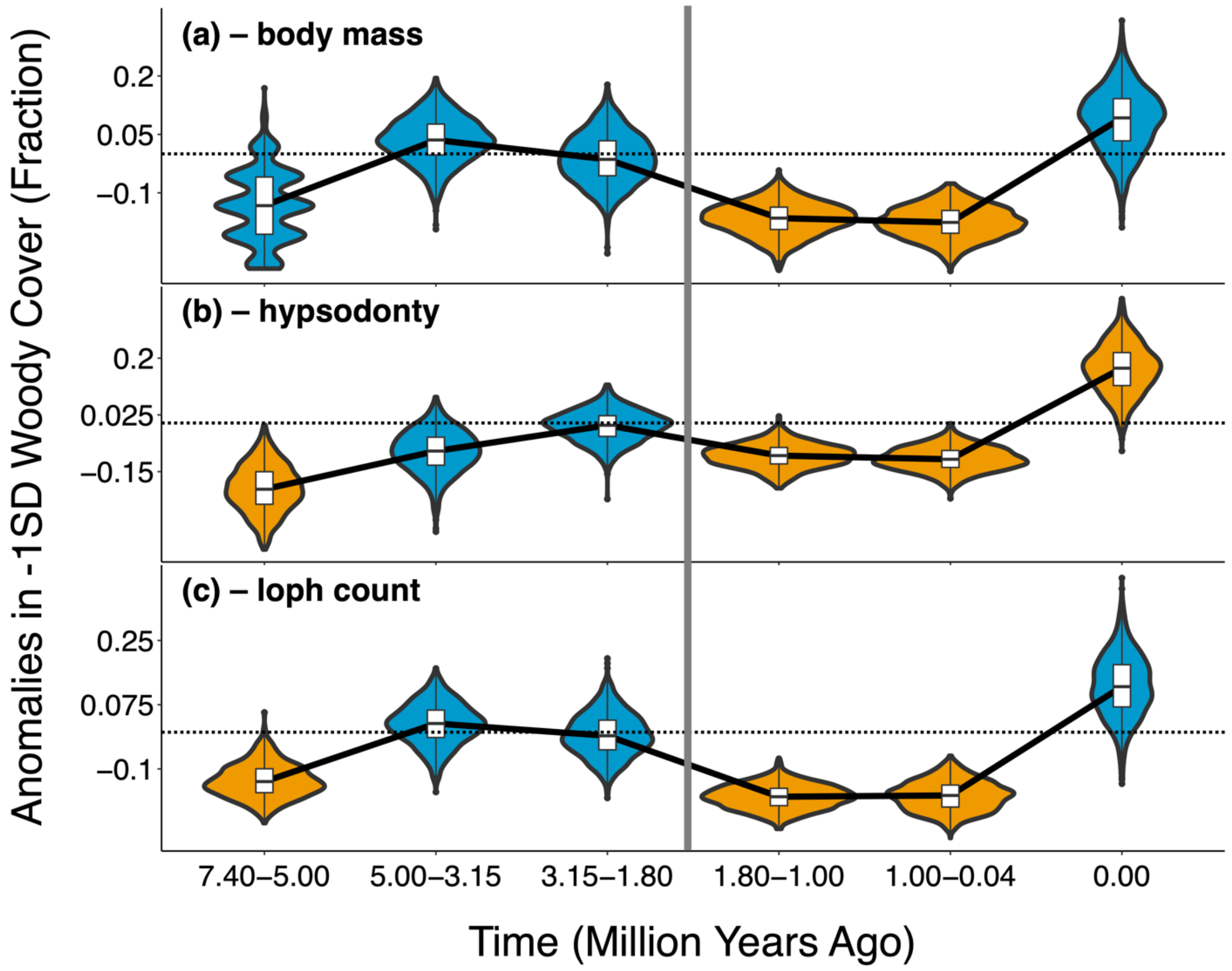

Supplementary Figure 7. Trends in the ecometric anomalies (see Supplementary Figure 1d) of eastern African communities of large herbivores, with respect to estimations of communities' mean - 1SD woody cover values from body mass (a), hypsodonty (b), and loph count (c). This figure differs from Figure 2 in that it refers to estimations of communities' woody cover values that are one standard deviation below their means (see Methods). Maximum likelihood estimations of woody cover were made for each community using an ecometric model of all communities together through time. Each violin plot depicts the distribution of n=1,000 independent samples, where each sample is the mean ecometric anomaly of a random subset of communities occurring within the plot's time bin (x-axis). Each box plot depicts its distribution's median (center) and interquartile range (bounds of box), plus 1.5 * the interquartile range above and below the box (whiskers). Dotted lines indicate a mean anomaly of zero. Blue plots represent distributions whose 95% confidence intervals contain zero, while orange plots represent those whose confidence intervals do not. The vertical gray line represents the point at which ecometric anomalies shifted significantly after a long period of consistency. Time bins on the x-axis are based on the cutoff points of the time intervals depicted in Figure 1. 3.15 Ma is the midpoint of 3.3-3 Ma, 1.8 Ma is the midpoint of 1.9-1.7 Ma, and the final time bin represents the present. Source data are provided as a Source Data file.

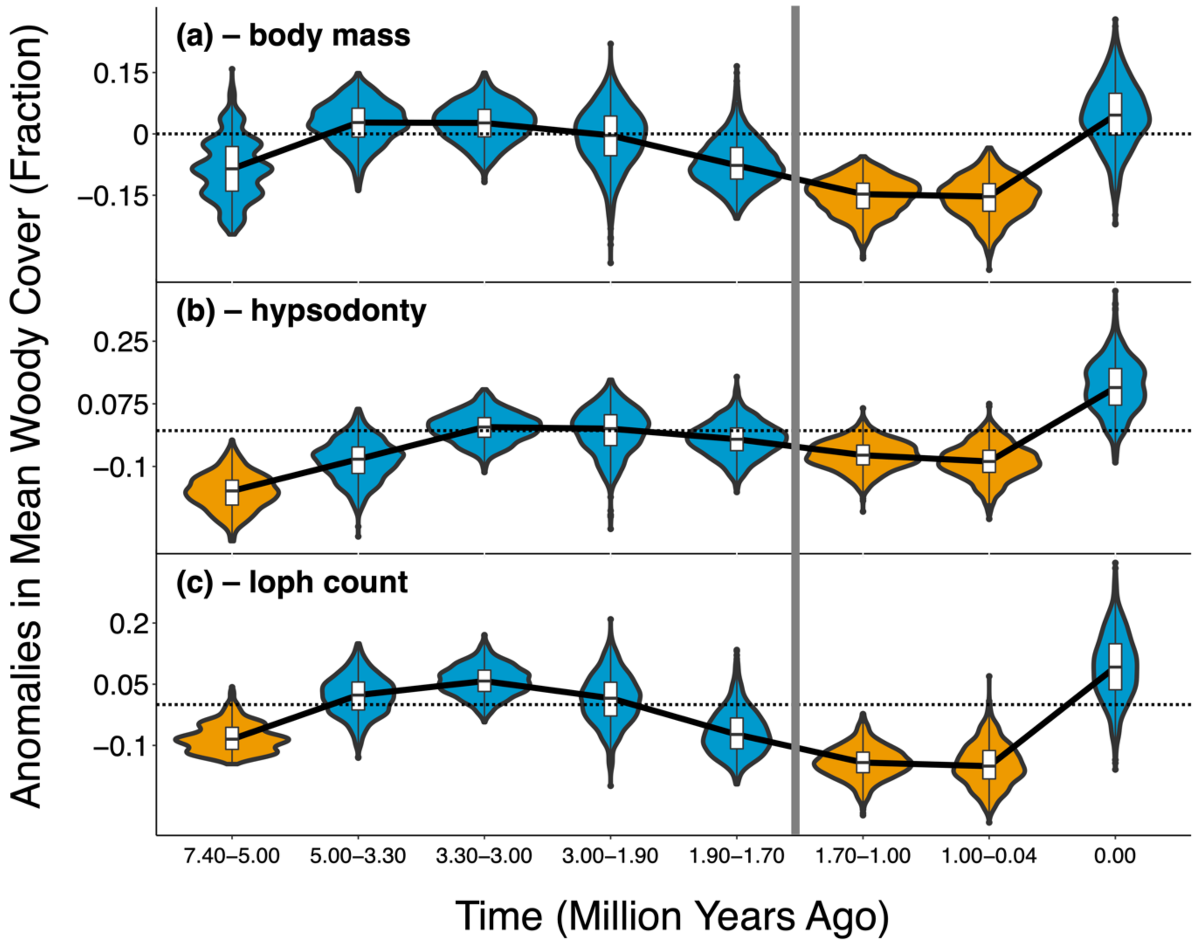

Supplementary Figure 8. Trends in the ecometric anomalies (see Supplementary Figure 1d) of eastern African communities of large herbivores, with respect to estimations of communities' mean woody cover values from body mass (a), hypsodonty (b), and loph count (c), using relatively fine-focused time bins. This figure differs from Figure 2 in that it includes time bins (x-axis) that focus in on 3.3-3 and 1.9-1.7 Ma, as opposed to using their midpoints of 3.15 and 1.8 Ma. Maximum likelihood estimations of woody cover were made for each community using an ecometric model of all communities together through time. Each violin plot depicts the distribution of n=1,000 independent samples, where each sample is the mean ecometric anomaly of a random subset of communities occurring within the plot's time bin (x-axis). Each box plot depicts its distribution's median (center) and interquartile range (bounds of box), plus 1.5 * the interquartile range above and below the box (whiskers). Dotted lines indicate a mean anomaly of zero. Blue plots represent distributions whose 95% confidence intervals contain zero, while orange plots represent those whose confidence intervals do not. The vertical gray line represents the point at which ecometric anomalies shifted significantly after a long period of consistency. Time bins on the x-axis are based on the cutoff points of the time intervals depicted in Figure 1, with the final time bin representing the present. Source data are provided as a Source Data file.

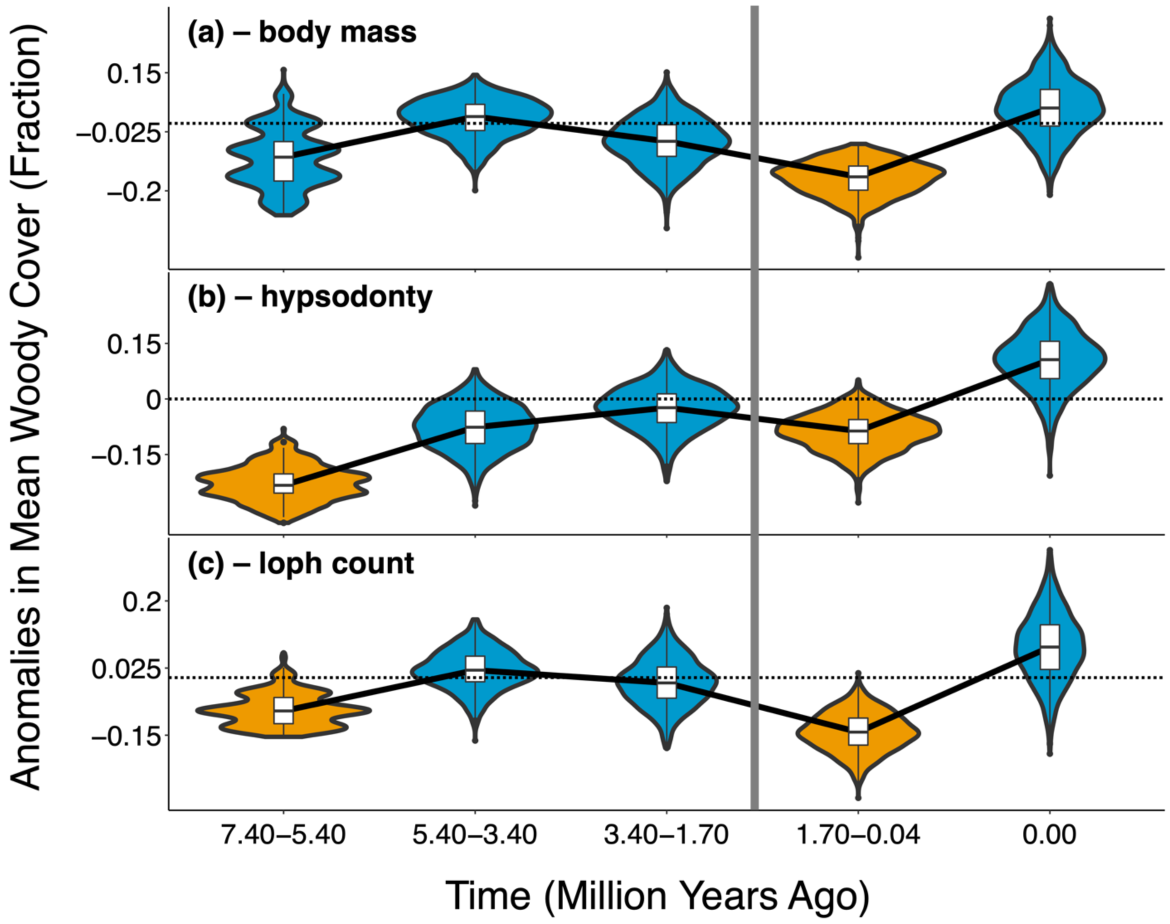

Supplementary Figure 9. Trends in the ecometric anomalies (see Supplementary Figure 1d) of eastern African communities of large herbivores, with respect to estimations of communities' mean woody cover values from body mass (a), hypsodonty (b), and loph count (c), using relatively uniform time bins. This figure differs from Figure 2 in that it includes time bins (x-axis) that are more uniform in size. Using bins that were identical in size, while also including a meaningful number of bins, was not feasible given the highly limited sample of sites occurring at earlier times (for example, only 8 sites occur within the first 3 Ma of our time series – from 7.4-4.4 Ma). We therefore used 2-Ma time bins for earlier times, and only slightly smaller 1.7-Ma bins for more recent times. The final time bin at 0 Ma represents the present. Maximum likelihood estimations of woody cover were made for each community using an ecometric model of all communities together through time. Each violin plot depicts the distribution of n=1,000 independent samples, where each sample is the mean ecometric anomaly of a random subset of communities occurring within the plot's time bin (x-axis). Each box plot depicts its distribution's median (center) and interquartile range (bounds of box), plus 1.5 * the interquartile range above and below the box (whiskers). Dotted lines indicate a mean anomaly of zero. Blue plots represent distributions whose 95% confidence intervals contain zero, while orange plots represent those whose confidence intervals do not. The vertical gray line represents the point at which ecometric anomalies shifted significantly after a long period of consistency. Source data are provided as a Source Data file.

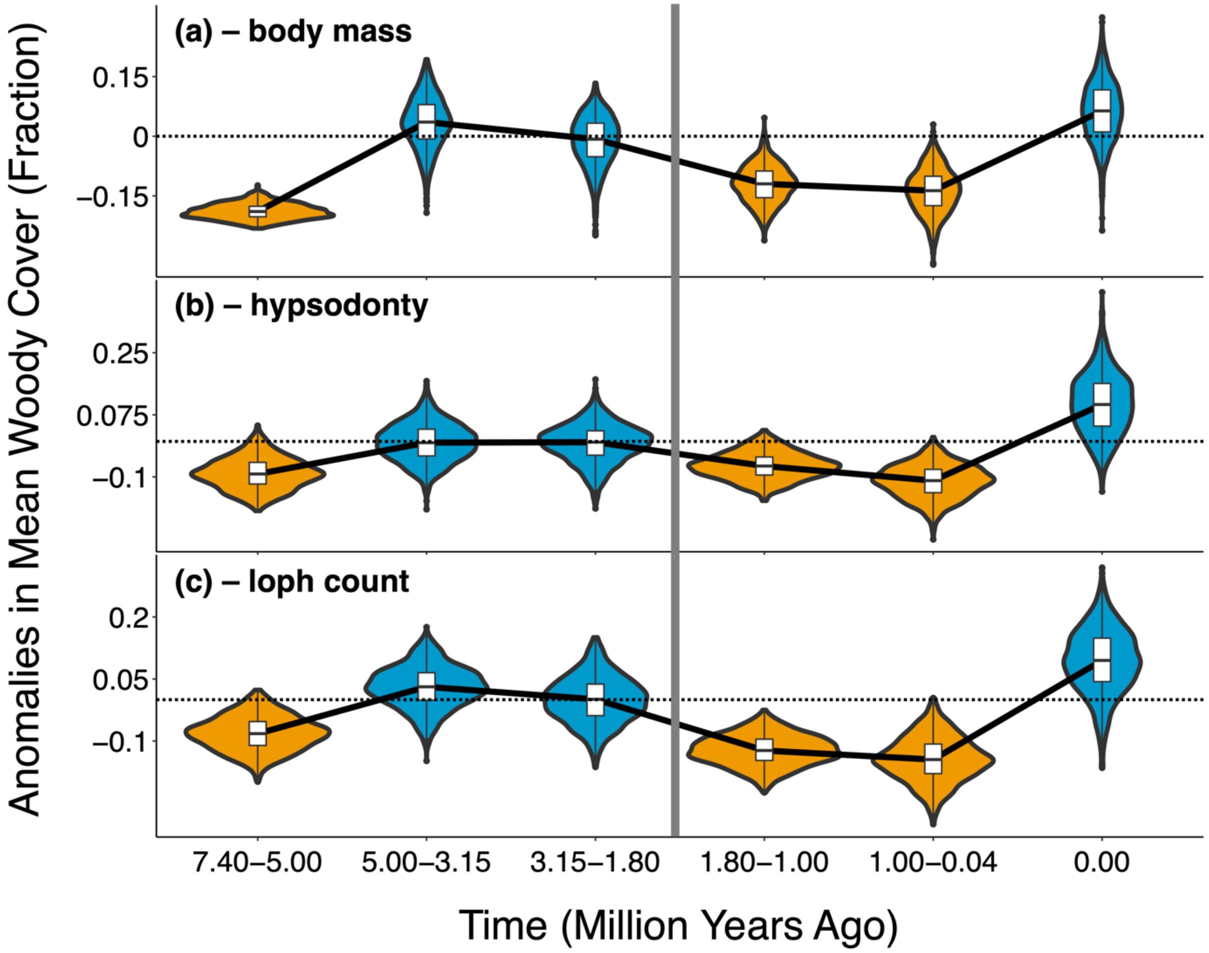

Supplementary Figure 10. Trends in the ecometric anomalies (see Supplementary Figure 1d) of eastern African communities of large herbivores (≥100 kg), with respect to estimations of communities' mean woody cover values from body mass (a), hypsodonty (b), and loph count (c). This figure differs from Figure 2 in that it only considers species that are ≥100 kg in mass, as opposed to ≥44 kg. Maximum likelihood estimations of woody cover were made for each community using an ecometric model of all communities together through time. Each violin plot depicts the distribution of n=1,000 independent samples, where each sample is the mean ecometric anomaly of a random subset of communities occurring within the plot's time bin (x-axis). Each box plot depicts its distribution's median (center) and interquartile range (bounds of box), plus 1.5 * the interquartile range above and below the box (whiskers). Dotted lines indicate a mean anomaly of zero. Blue plots represent distributions whose 95% confidence intervals contain zero, while orange plots represent those whose confidence intervals do not. The vertical gray line represents the point at which ecometric anomalies shifted significantly after a long period of consistency. Time bins on the x-axis are based on the cutoff points of the time intervals depicted in Figure 1. 3.15 Ma is the midpoint of 3.3-3 Ma, 1.8 Ma is the midpoint of 1.9-1.7 Ma, and the final time bin represents the present. Source data are provided as a Source Data file.

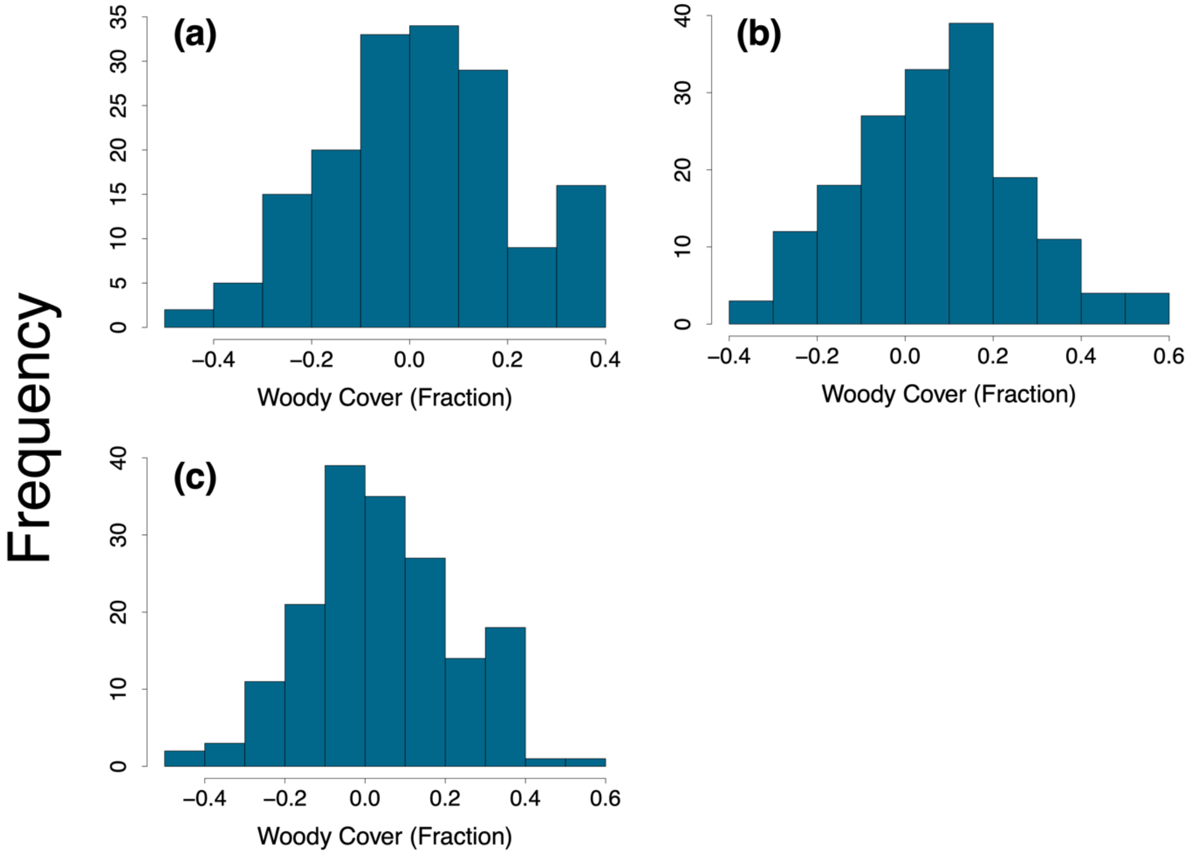

Supplementary Figure 11. Distributions of the ecometric anomalies of eastern African communities of large herbivores, stemming from ecometric models that address body mass (a), hypsodonty (b), and loph count (c). For a given community, an ecometric anomaly is calculated as its measured minus its maximum-likelihood-estimated fraction of woody cover. In all models, the greatest frequencies of anomalies are near zero, and high-magnitude anomalies are relatively infrequent. This suggests reliable estimates of woody cover from traits [2]. Source data are provided as a Source Data file.

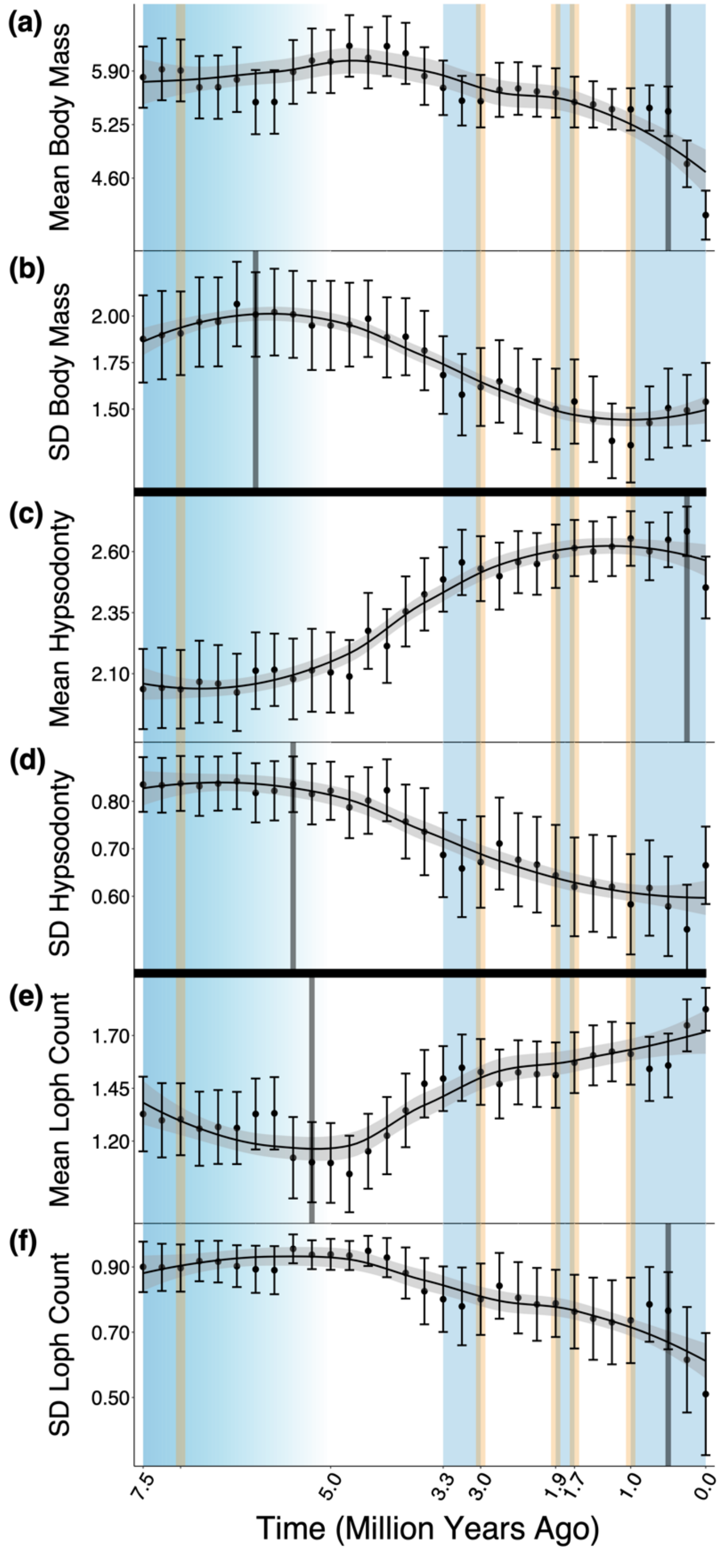

Supplementary Figure 12. LOESS regression trends in the mean and standard deviation of body mass (a-b), hypsodonty (c-d), and longitudinal loph count (e-f) in all eastern African herbivores (megafauna ≥44 kg in mass and non-megafauna <44 kg). Trends are nearly identical to those in Figure 3. Each data point represents a 250,000-year time bin. Body mass is measured in log-transformed kg, hypsodonty on a discrete scale of 1 to 3 (1 = brachydont, 2 = mesodont, 3 = hypsodont), and loph count as a count from 0 to 2. Each data point is presented as a mean value ± one standard error from n=1,000 independent samples of species (see Methods). LOESS regression curves use a smoothing parameter of 0.75. Faded gray bars denote breakpoints from breakpoint analysis. Blue shaded areas refer to events related to environmental change and orange to events in hominin evolution. These events are encompassed in key time intervals, as follows: 7.5-5 Ma includes the onset of grassland expansion and the emergence of hominins (7 Ma); 3.3-3 Ma includes the mid-Pliocene Warm Period and the development of Oldowan hominin tools (3 Ma); 1.9-1.7 Ma includes the increase in climate variability and aridity, as well as the emergence of *Homo erectus* (1.9 Ma) and their development of Acheulean technology (1.7 Ma); and ≤1 Ma includes the intensification of periods of aridity, as well as rapid cranial growth in hominins (1 Ma). Source data are provided as a Source Data file.

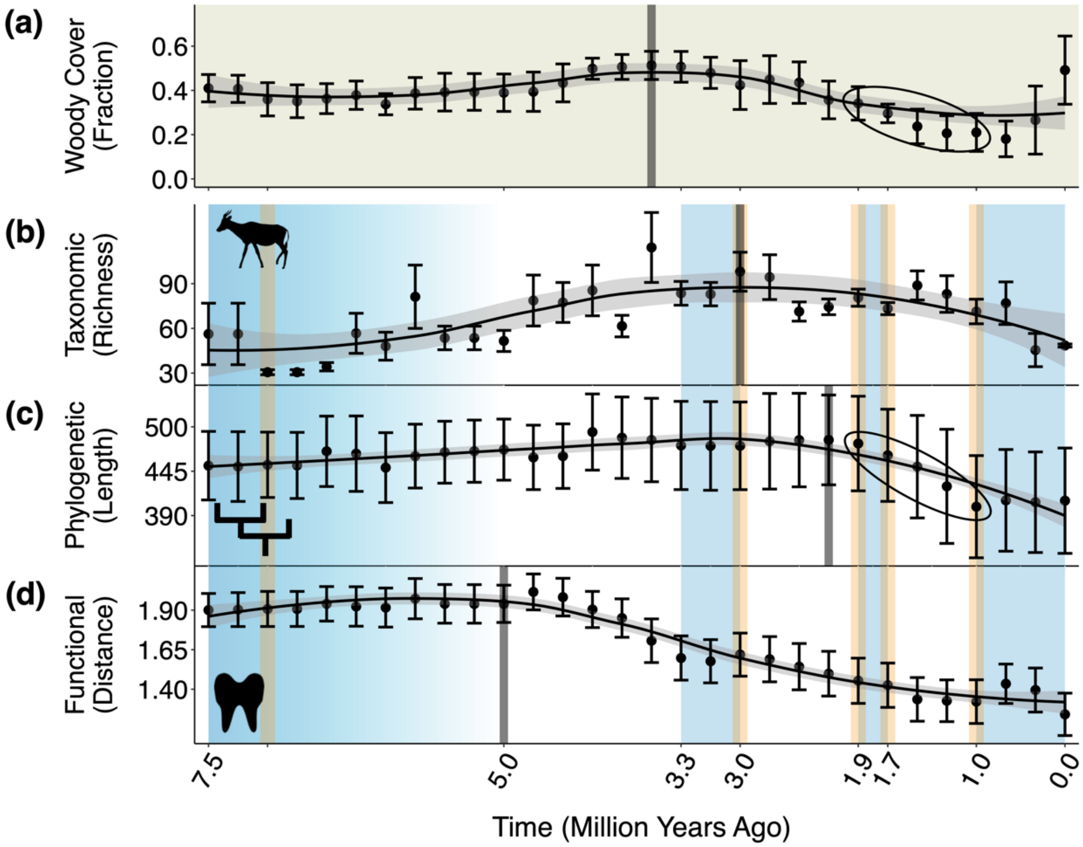

Supplementary Figure 13. LOESS regression trends in the fraction of woody cover among communities of herbivorous megafauna in eastern Africa (a), as well as in megafaunal taxonomic (b), phylogenetic (c), and functional diversity (d). This figure differs from Figure 1 in that each data point refers not to a sequential 250,000-year time bin, but to a 500,000-year overlapping bin (7.5-7, 7.25-6.75, … Ma). Trends are nearly identical to those in Figure 1, indicating that our choice of time bins did not impact our results. Among the species of megafauna occurring in each time bin, taxonomic diversity is measured as species richness; phylogenetic diversity as the sum of the branch lengths (in millions of years) of the phylogenetic tree connecting genera; and functional diversity as the mean Euclidean distance between species' trait values (body mass, hypsodonty, and loph count) and their centroid in three-dimensional space (see Supplementary Figure 4). Each data point is presented as a mean value $\pm$ one standard error from n=1,000 independent samples of communities (a), species occurrences (b), genera (c), or species (d) (see Methods). LOESS regression curves use a smoothing parameter of 0.75. Faded gray bars denote breakpoints from breakpoint analysis. Blue shaded areas refer to events related to environmental change and orange to events in hominin evolution. These events are encompassed in key time intervals, as follows: 7.5-5 Ma includes the onset of grassland expansion and the emergence of hominins (7 Ma); 3.3-3 Ma includes the mid-Pliocene Warm Period and the development of Oldowan hominin tools (3 Ma); 1.9-1.7 Ma includes the increase in climate variability and aridity, as well as the emergence of *Homo erectus* (1.9 Ma) and their development of Acheulean technology (1.7 Ma); and $\leq$1 Ma includes the intensification of periods of aridity, as well as rapid cranial growth in hominins (1 Ma). Source data are provided as a Source Data file.

Supplementary References

1 Faith, J. T., Rowan, J. & Du, A. Early hominins evolved within non-analog ecosystems. *Proceedings of the National Academy of Sciences* **116**, 21478-21483 (2019).

2 Short, R. A., Pinson, K. & Lawing, A. M. Comparison of environmental inference approaches for ecometric analyses: Using hypsodonty to estimate precipitation. *Ecology and Evolution* **11**, 587-598 (2021).
