# Supplementary material for: Disruption of trait-environment relationships in African megafauna occurred in the middle Pleistocene
Source: Nat Commun. 2023 Jul 18;14:4016. doi: 10.1038/s41467-023-39480-8 (PMC10354096; doi:10.1038/s41467-023-39480-8)
Supplement: Supplementary file 3 — Description of Additional Supplementary Files [file 41467_2023_39480_MOESM3_ESM.pdf]

Description of Additional Supplementary Files for

Disruption of trait-environment relationships in African megafauna occurred in the middle Pleistocene

Daniel A. Lauer*, A. Michelle Lawing, Rachel A. Short, Fredrick K. Manthi, Johannes Müller, Jason J. Head, Jenny L. McGuire

*Daniel A. Lauer
E-mail: lauerd@gatech.edu

Supplementary Data Legends

Supplementary Data 1. (Separate file). Data of the 203 fossil species analyzed in this study. Information about the species in the dataset includes their taxonomic identifications, body masses, dental traits, minimum and maximum ages of occurrence, and occurrences at or absences from each of 58 fossil sites. Species shaded in gray are not included in the count of 203 species: they were removed from all analyses except for in the production of Supplementary Figure 12, as they refer to species that are <44 kg in mass (see Methods). Sites shaded in gray are not included in the count of 58 sites and were not analyzed: they either are not associated with a temporal range of occurrence, or quantitative estimates of their woody cover are not available.

Supplementary Data 2. (Separate file). Data of the 48 modern species analyzed in this study. Information about the species in the dataset includes their taxonomic identifications, body masses, dental traits, and occurrences at or absences from each of 128 modern sites. Species shaded in gray are not included in the count of 48 species: they were removed from all analyses except for in the production of Supplementary Figure 12, as they refer to species that are <44 kg in mass (see Methods). Sites shaded in gray are not included in the count of 128 sites and were not analyzed, because quantitative estimates of their woody cover are not available.

Supplementary Data 3. (Separate file). Data of the fraction of woody cover for each of the fossil sites analyzed in this study. Information about each site includes its age range, mean and standard deviation fraction of woody cover, and relevant citations.

Supplementary Data 4. (Separate file). Data of the fraction of woody cover for each of the modern sites analyzed in this study. These data were obtained directly from the literature [1].

Reference

1 Barr, W. A. & Biernat, M. Mammal functional diversity and habitat heterogeneity: Implications for hominin habitat reconstruction. *Journal of Human Evolution* **146**, 102853 (2020).
