# Supplementary material for: Disruption of trait-environment relationships in African megafauna occurred in the middle Pleistocene
Source: Nat Commun. 2023 Jul 18;14:4016. doi: 10.1038/s41467-023-39480-8 (PMC10354096; doi:10.1038/s41467-023-39480-8)
Supplement: Supplementary file 8 — Reporting Summary [file 41467_2023_39480_MOESM8_ESM.pdf]

nature portfolio

Corresponding author(s): Daniel Avery Lauer

Last updated by author(s): Jun 8, 2023

Reporting Summary

Nature Portfolio wishes to improve the reproducibility of the work that we publish. This form provides structure for consistency and transparency in reporting. For further information on Nature Portfolio policies, see our Editorial Policies and the Editorial Policy Checklist.

Statistics

For all statistical analyses, confirm that the following items are present in the figure legend, table legend, main text, or Methods section.

n/a	Confirmed	
☐	☒	The exact sample size (*n*) for each experimental group/condition, given as a discrete number and unit of measurement
☐	☒	A statement on whether measurements were taken from distinct samples or whether the same sample was measured repeatedly
☐	☒	The statistical test(s) used AND whether they are one- or two-sided<br>*Only common tests should be described solely by name; describe more complex techniques in the Methods section.*
☐	☒	A description of all covariates tested
☐	☒	A description of any assumptions or corrections, such as tests of normality and adjustment for multiple comparisons
☐	☒	A full description of the statistical parameters including central tendency (e.g. means) or other basic estimates (e.g. regression coefficient) AND variation (e.g. standard deviation) or associated estimates of uncertainty (e.g. confidence intervals)
☐	☒	For null hypothesis testing, the test statistic (e.g. *F*, *t*, *r*) with confidence intervals, effect sizes, degrees of freedom and *P* value noted<br>*Give P values as exact values whenever suitable.*
☒	☐	For Bayesian analysis, information on the choice of priors and Markov chain Monte Carlo settings
☒	☐	For hierarchical and complex designs, identification of the appropriate level for tests and full reporting of outcomes
☐	☒	Estimates of effect sizes (e.g. Cohen's *d*, Pearson's *r*), indicating how they were calculated

Our web collection on statistics for biologists contains articles on many of the points above.

Software and code

Policy information about availability of computer code

Data collection	The R code written to collect and process data, and the files on which that code depends, are available on GitHub at https://github.com/lauerd/MegafaunaEcometrics.
Data analysis	The R code written to conduct all analyses, and the files on which that code depends, are available on GitHub at https://github.com/lauerd/MegafaunaEcometrics.

For manuscripts utilizing custom algorithms or software that are central to the research but not yet described in published literature, software must be made available to editors and reviewers. We strongly encourage code deposition in a community repository (e.g. GitHub). See the Nature Portfolio guidelines for submitting code & software for further information.

Data

Policy information about availability of data

All manuscripts must include a data availability statement. This statement should provide the following information, where applicable:

- Accession codes, unique identifiers, or web links for publicly available datasets
- A description of any restrictions on data availability
- For clinical datasets or third party data, please ensure that the statement adheres to our policy

The data generated in this study are provided in the Supplementary Information and Source Data file. The Supplementary Information includes Supplementary Datasets 1-4, which are available as Microsoft Excel files. The legends for these datasets, which describe the datasets in detail, can be found in the Description of Additional Supplementary Files file.

Human research participants

Policy information about studies involving human research participants and Sex and Gender in Research.

Reporting on sex and gender	Not applicable.
Population characteristics	Not applicable.
Recruitment	Not applicable.
Ethics oversight	Not applicable.

Note that full information on the approval of the study protocol must also be provided in the manuscript.

Field-specific reporting

Please select the one below that is the best fit for your research. If you are not sure, read the appropriate sections before making your selection.

☐ Life sciences ☐ Behavioural & social sciences ☒ Ecological, evolutionary & environmental sciences

For a reference copy of the document with all sections, see nature.com/documents/nr-reporting-summary-flat.pdf

Ecological, evolutionary & environmental sciences study design

All studies must disclose on these points even when the disclosure is negative.

Study description	Our study investigated if and when any disruptions occurred in the functional trait-environment relationships of herbivorous, eastern African megafauna over the past 7.4 Ma (a period of dramatic environmental change and hominin evolution). We determined if such disruptions were coincident with any losses in megafaunal biodiversity. Thus, we performed time-series analyses to determine how megafaunal biodiversity and trait-environment relationships changed over time (see sample sizes for these analyses below).
Research sample	We analyzed a previously published compilation of the occurrences of mammalian herbivore species (orders Artiodactyla, Perissodactyla, Proboscidea) across African sites. The compilation was made in a 2018 study of African megaherbivore extinctions from 7.4 Ma to the present (Faith et al., 2018). It includes 349 species at 101 fossil sites and 89 species at 203 modern sites. Using a series of criteria related to data quality and relevance, we analyzed 203 of the fossil species and 48 of the modern species. These species are a robust representation of the available record of large, herbivorous mammals that have occurred in Africa since the Plio-Pleistocene.<br><br>Reference: Faith, J. T., Rowan, J., Du, A. & Koch, P. L. Plio-Pleistocene decline of African megaherbivores: No evidence for ancient hominin impacts. Science 362, 938-941 (2018).
Sampling strategy	No sample size calculation was performed, as the sample size in our dataset was determined by the number of species in the Faith et al., 2018 dataset (see above). The 203 fossil and 48 modern species that we analyzed form a strong representation of the herbivorous megafauna that have occurred in Africa over the late Cenozoic.
Data collection	Data representing the herbivore species' occurrences, functional traits, phylogenies, and site-level environmental conditions were compiled by the corresponding author from a variety of primary literature articles and publicly available data sources.
Timing and spatial scale	The data in the Faith et al., 2018 dataset (see above) span the past 7.4 Ma. While the sites at which herbivore species occur/occurred are unevenly distributed over time, we applied a series of methods to account for temporal sampling bias. Herbivore occurrences were recorded previously (in Faith et al., 2018 and the references therein) across various archaeological sites.
Data exclusions	We excluded herbivore species that were associated with vague taxonomic identifications or that were too small in body mass to be considered megafauna (<44 kg). We also excluded species that occurred only at sites without known age ranges and/or without available quantitative estimates of their fraction of woody cover (the environmental condition of interest).
Reproducibility	All R code needed to reproduce the findings of this study are publicly available in GitHub (https://github.com/lauerd/MegafaunaEcometrics). Additionally, we performed a series of sensitivity analyses throughout our study to ensure the robustness and reproducibility of our results.
Randomization	Herbivore species were divided into groups based on the time periods at which they occurred. This was necessary to perform time-series analyses of biodiversity patterns and trait-environment relationships over the past 7.4 Ma.
Blinding	Blinding was not relevant to our study. We collected and analyzed data from publicly available data sources, and we used an analytical/computational (as opposed to experimental) study approach that does not require any form of blinding to ensure the validity of our results.

Did the study involve field work? ☐ Yes ☒ No

Reporting for specific materials, systems and methods

We require information from authors about some types of materials, experimental systems and methods used in many studies. Here, indicate whether each material, system or method listed is relevant to your study. If you are not sure if a list item applies to your research, read the appropriate section before selecting a response.

Materials & experimental systems

n/a	Involved in the study
☒	☐ Antibodies
☒	☐ Eukaryotic cell lines
☒	☐ Palaeontology and archaeology
☒	☐ Animals and other organisms
☒	☐ Clinical data
☒	☐ Dual use research of concern

Methods

n/a	Involved in the study
☒	☐ ChIP-seq
☒	☐ Flow cytometry
☒	☐ MRI-based neuroimaging
